# Illness perceptions, risk perceptions and worries in patients with early systemic sclerosis: A focus group study

**DOI:** 10.1002/msc.1453

**Published:** 2020-01-26

**Authors:** Nina M. van Leeuwen, Maaike Boonstra, Tom W.J. Huizinga, Ad A. Kaptein, Jeska K. de Vries‐Bouwstra

**Affiliations:** ^1^ Department of Rheumatology Leiden University Medical Centre Leiden The Netherlands; ^2^ Department of Rheumatology Amsterdam University Medical Centre Amsterdam The Netherlands; ^3^ Department of Medical Psychology Leiden University Medical Centre Leiden The Netherlands

**Keywords:** systemic sclerosis, focus group, drawings, illness perceptions, patients' view

## Abstract

**Objectives:**

This study explores illness perceptions, risk perceptions and degree of worry in patients with recently diagnosed systemic sclerosis (SSc). Specifically, it aims to answer whether and how early diagnosis in a stage that disease is relatively mild can impact patients' lives, and if and how disease severity associates with illness perceptions and risk perception.

**Methods:**

Patients with a diagnosis of SSc <2 years were invited to participate in a focus group discussion for in‐depth exploration of illness perceptions, risk perceptions and worry. In addition, illness perceptions, risk perceptions and worries were evaluated with the use of questionnaires. To explore how patients perceive SSc, we asked them to draw their disease. Physician global assessment of disease severity was used to measure disease severity. Associations between disease severity, illness/risk perceptions, drawings and elements of the focus group were assessed.

**Results:**

We observed three dimensions of illness perception as most relevant for patients: personal control, concern and consequences. Patients with SSc experienced many symptoms and felt low personal control. Concerns about the future were often mentioned, and the majority of patients scored high on the worry questionnaire. None of the patients was preoccupied with prognosis or death. All drawings illustrate the impact of SSc on daily life and psychological well‐being. Illness perceptions were highly variable between patients and did not associate with disease severity.

**Conclusion:**

This study showed that a diagnosis of early SSc had a significant impact on patients' lives, also in the absence of severe disease complications.

## 1 INTRODUCTION

Systemic sclerosis (SSc) is a chronic and incurable connective tissue disease with a heterogeneous presentation and disease course (Allanore et al., 2015). Skin fibrosis is characteristic, but interstitial lung disease (ILD), peripheral vasculopathy and gastrointestinal involvement are also common. More severe disease complications such as myocardial disease and pulmonary arterial hypertension, although less frequent, are associated with increased mortality (Elhai et al., 2017) and require monitoring. The first 5 years of the disease are the most critical in determining an individual patient's prognosis (Khanna & Denton, 2010). With the improvement of diagnostic guidelines, the diagnosis is more frequently made during an early phase (Vasile et al., 2018). However, earlier diagnosis has not led to improvements in determining the prognosis in the individual patient due to lack of accurate prognostic markers and that early diagnosis lengthens the time‐window in which a prognosis is unclear (Minier et al., 2014). It is important and helpful to know how this affects patients, as illness perceptions directly influence illness behaviour (Amin et al., 2011; Merz et al., 2017). However, the patient's perception of risk for severe complications of SSc has hardly been studied.

Several studies indicate that among persons with chronic illnesses the subjective beliefs about their condition are strongly associated with outcomes such as pain, physical health status and mental health status (Brekke, Hjortdahl, & Kvien, 2003; Chiang, Bundy, Griffiths, Paus, & Harries, 2015; Jopson & Moss‐Morris, 2003; Ndosi et al., 2016; Reynolds, Broadbent, Ellis, Gamble, & Petrie, 2007). Beliefs regarding the patient's condition are referred to as illness perceptions. These comprise the patient's own ideas about the disease, its cause, how the disease evolves over time, what the consequences will be, how the disease should be treated, and emotional responses to the illness and its consequences. Previous evaluation of illness perceptions in longstanding SSc showed that patients generally held strong views about the chronic nature and negative consequences of the disease (Nakayama et al., 2016; Sumpton et al., 2017). The unpredictable disease course and being at risk for organ involvement were found as important areas of illness perceptions (Frantz et al., 2016; Nakayama et al., 2016; Sumpton et al., 2017). Interestingly, illness perceptions contributed more to physical and mental health in SSc than disease variables commonly used to describe disease severity (Arat et al., 2012; Richards et al., 2003).

Risk perceptions comprise the result of individual patient characteristics, including coping strategies, in combination with illness perceptions. These risk perceptions have a major impact on level of worry (Ferrer & Klein, 2015), illness behaviour (e.g., adherence with medication, seeking help from healthcare providers, refraining from work, and sexual activities) and commitment to medical care, which in turn affects the outcome of the illness and its medical management (Cameron & Diefenbach, 2001; Kemp, Morley, & Anderson, 1999). However, in‐depth interviews on these issues in early SSc are lacking. The traditional method to elucidate patients' illness and risk perceptions includes questionnaires and focus group interviews. Although of value, these methods might influence patients' answers due to social desirability. A personal drawing of the disease can illustrate the psychological and social impact of the disease of the individual patient and can reveal issues that remain unspoken during focus group discussions because of patient embarrassment, stigma and shame (Broadbent, Schoones, Tiemensma, & Kaptein, 2019). Indeed, a recent review revealed that drawings supplement and potentially outperform traditional data collection approaches (Broadbent et al., 2019).

In the current study, we explored illness perception, risk perception and degree of worry in patients with recently diagnosed SSc, when prognosis is still uncertain. We performed an explorative, in‐depth study combining quantitative measures such as questionnaires, qualitative measures such as a focus group, and individual drawings in a selected group of patients with SSc.

## 2 METHODS

### 2.1 Participants

Patients from the Combined Care Pathway in Systemic Sclerosis (CCISS) (Meijs et al., 2016) were eligible for inclusion. This is an observational cohort of patients with SSc, with annual follow‐up in the Department of Rheumatology at the Leiden University Medical Centre (LUMC). Following written informed consent, patient data are collected systematically, including results of physical examination and extensive screening for organ involvement. For the current study, we selected patients aged 18–60 years that had received the diagnosis of SSc according to ACR/EULAR 2013 criteria (American College of Rheumatology/European League against Rheumatism) (van den Hoogen et al., 2013) between 1 and 2 years before the start of this study. This time frame was chosen to allow patients with recently diagnosed SSc an appropriate amount of time to develop personal illness/risk perceptions, while excluding patients with well‐established disease (>2 years) that might have already developed severe disease‐related morbidity and in whom it was not possible to assess future risk perceptions. For that same reason, we excluded patients with severe organ involvement requiring stem cell transplantation and/or end‐stage organ involvement. In addition, patients had to have completed a second evaluation in the care programme and started with any kind of medication (prescribed by the rheumatologist) because of SSc. Patients with a psychiatric medical history were excluded.

### 2.2 Brief Illness Perception Questionnaire

Illness perceptions were assessed using the Brief Illness Perception Questionnaire (BIPQ; Broadbent et al., 2015). The BIPQ consists of nine questions: (1) perceived consequences; (2) timeline (acute‐chronic); (3) amount of perceived personal control; (4) treatment control; (5) identity (symptoms); (6) concern about the disease; (7) coherence of the illness; (8) emotional representation; and (9) causal perception. Items 6 and 8 overlap, with an assessment of concern about the illness and assessment of the emotional aspects and mood of patients. Item 9 allows the patient to give three factors that in his/her opinion have caused the disease. Each item is rated on a 10‐point scale, where higher scores in questions 1, 2, 5, 6 and 8 represent stronger negative endorsement with the illness perception. Higher scores in questions 3, 4 and 7 represent positive endorsement with that perception.

### 2.3 Risk perceptions and worry

Perceived risks of disease complications, intensive treatment and death were assessed using the adapted questionnaire from Cameron and Diefenbach (2001), consisting of three questions each with two subquestions:

*1.1) How likely do you think it is that, at some point in your life, you will get a disease complication that will influence your way of life?, 1.2) How vulnerable do you think you are to develop a disease complication that will influence your way of life, at some point in your life?; 2.1) How likely do you think you are to get a disease complication that requires intensive treatment such as chemotherapy (cyclophosphamide) or stem cell transplantation, at some point in your life?, 2.2) How vulnerable do you think you are to develop a disease complication that requires intensive treatment such as chemotherapy (cyclophosphamide) or stem cell transplantation, at some point in your life?; 3.1) How likely do you think it is that, at some point in your life, you will get a disease complication that will result in death?, 3.2) How vulnerable do you think you are to develop a disease complication that will result in death, at some point in your life?.*

Each item is rated on a seven‐point Likert scale ranging from 1 (not at all) to 7 (almost certain or extremely). To calculate scores for risk perception, ratings of subscores were added (range 2–14) for each pair of questions.

Perceived worry was assessed with the following questions, also adapted from Cameron and Diefenbach (2001): “1. To what extent are you worried about the disease worsening?” and “2. To what extent are you concerned about the disease worsening?”. These items were also rated on a seven‐point Likert scale ranging from 1 (not at all) to 7 (almost certain or extremely). Addition of the two questions generated a total worry score (range 2–14).

### 2.4 Focus group and drawings

**Figure 3:**
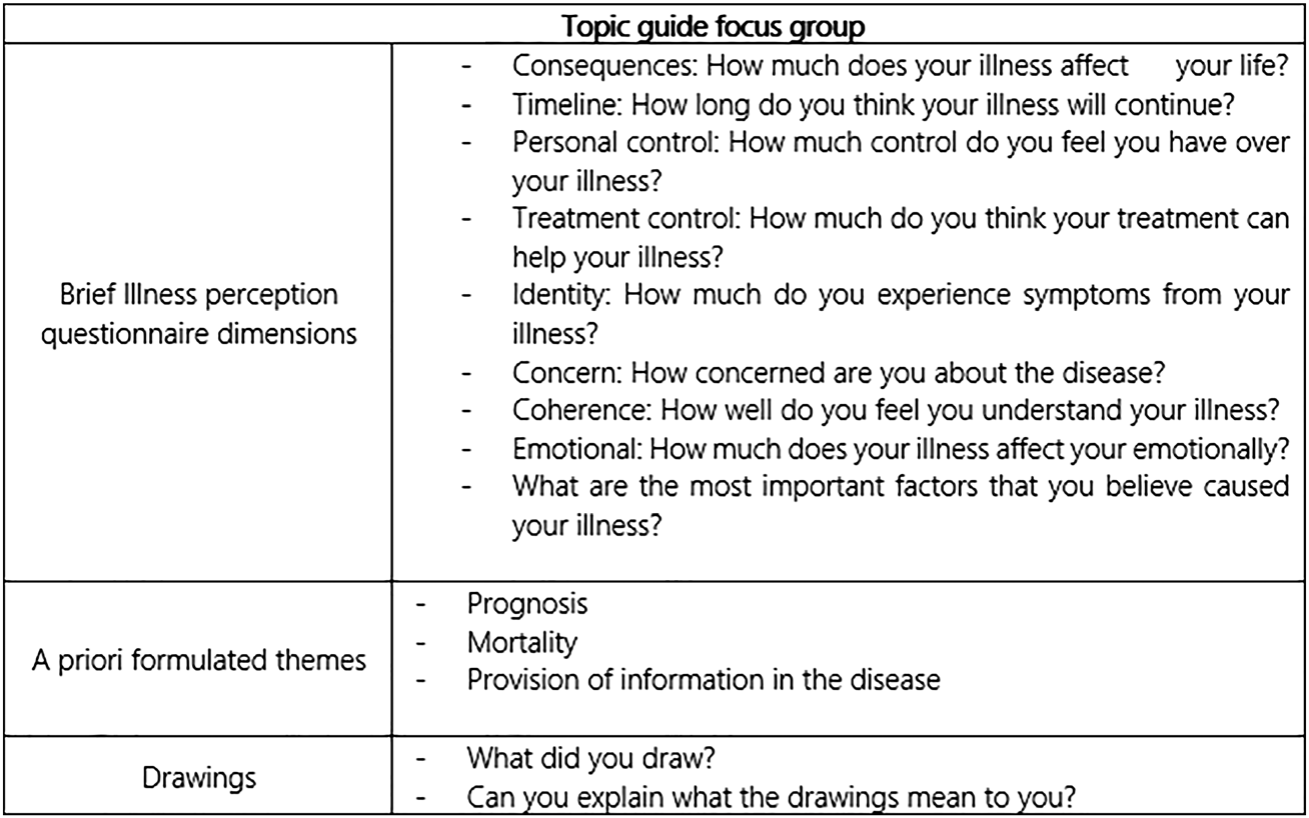
Topic guide focus group

A focus group discussion was held in an informal setting in a meeting room of the LUMC (outside the outpatient clinic) with coffee, tea and biscuits, and lasted 2 h. The discussion was chaired by a health psychologist experienced in group discussions (A.A.K.), one researcher (N.v.L.) and one rheumatologist (J.d.V.B.) observed the meeting. Audio of the discussion was recorded and transcribed verbatim. Focus group discussions are valuable because discussions between patients indicate not only what patients think, but also how they think and why they think that way (Kitzinger, 1995). A focus group generates rich narrative data that provides in‐depth insights into patient perspectives on living with SSc. The optimal size for a focus group is between four and 12 participants; we included nine (four cancellations). This sample size created a large enough group to facilitate discussion without inhibiting balanced participation. Having a homogeneous group facilitates a narrative of shared experiences, fosters group comfort and cohesion, and improves the quality of group interaction (Dilorio, Hockenberry‐Eaton, Maibach, & Rivero, 1994; Doria et al., 2018). The study was designed in accordance with suggestions from the patient board of the Department of Rheumatology of the LUMC. Patients with different rheumatic disease, including two patients with SSc, take part on this board and are involved in research as performed by members of the department. The rheumatologist involved in SSc (J.d.V.R.) proposed to investigate the impact of prognosis and the value of biomarkers from a patients' perspective in SSc during one of the board meetings. The a priori themes evolved out of discussions with the rheumatologist specialized in SSc and a medical psychologist, and included prognosis, mortality and information on the disease (Figure 3). Two patients with SSc participating in the CCISS cohort of the LUMC were involved in the development of the focus group discussion and evaluated the questionnaires. Before the interview, all patients were asked to complete the questionnaires and make a drawing representing their SSc. No further instructions were given regarding the drawing, and patients were not asked to draw a specific organ or whatsoever. Patients were asked to provide a brief written explanation of their drawing to make its content more readily identifiable (Broadbent et al., 2019). The dimensions of the BIPQ, the drawings and a priori formulated themes were used as a guideline during the focus group discussion (Figure 3). Patients were invited to discuss further issues that had not been brought up but that they felt were important too.

### 2.5 Disease severity

Disease severity was evaluated using the physician global assessment tool, measured with a 0–100 mm visual analogue scale (VAS; scale 0–100) (Medsger et al., 2003; Steen & Medsger, 1997). VAS is a score to evaluate SSc organ system symptoms, including Raynaud's phenomenon, gastrointestinal tract, cardiac and lung involvement, pain and overall disease severity (Medsger et al., 2003; Steen & Medsger, 1997). All scores were given by the same physician (J.d.V.B.), as this physician was the treating rheumatologist for all included patients. A higher score indicates a more severe disease.

### 2.6 Analysis

Because of the small sample size, statistical testing and formal correlation analyses were not possible. Instead, summary scores and within‐patient relationships between dimensions were analysed. Illness perceptions were assessed with the BIPQ during the focus group and in the drawings. Risk perceptions and worry were assessed in the questionnaires and during the focus group. Mean scores on the BIPQ, risk perception and worry are presented. The relationship between BIPQ, risk perception, worry questionnaires and the drawings were evaluated. Per patient, we explored the association between illness/risk perceptions as measured by the questionnaire and disease severity, as measured by the physical global assessment tool for disease severity (VAS score). Individual stories of patients in the focus group transcript were analysed using interpretative phenomenological analysis (Smith, 2011) by two researchers (A.A.K. and N.v.L.) independently, and coded according to the dimensions of the BIPQ. The dimensions used for coding were perceived consequences, timeline (acute‐chronic), amount of perceived personal control, treatment control, identity (symptoms), concern about the disease, coherence of the illness and emotional representation. These dimensions were also used to code the drawings. Differences in coding between the two researchers were discussed with the third researcher (J.d.V.B.) until consensus was achieved. Characteristics of patients were analysed using SPSS software (IBM Corp., Armonk, NY).

### 2.7 Ethical approval

Ethical approval for data collection in this cohort was obtained from the Institutional Review Board of the LUMC (P18.200) and signed informed consent was obtained from all participants.

## 3 RESULTS

### 3.1 Characteristics of participants

Of the 23 persons with recently diagnosed SSc that were approached, nine agreed to participate in the focus group discussion. Unfortunately, four had to cancel the focus group due to illness (two), a car accident (one) and anxiety related to the meeting (one) on the day the discussion was scheduled. Of these four, two did complete the questionnaire and made a drawing. The clinical characteristics of the seven patients with complete or partial data are summarized in Table 1.

**Table 1:** Baseline characteristics

	Sex	Age	Time since onset Raynaud	Time since onset non‐Raynaud	Disease subset	Pitting scars	Digital ulcers	mRSS	ILD	PAH, cardiac involvement, renal crisis	SSc‐specific autoantibody	VAS score	Immunotherapy	Drawing
P1	F	43	1.5	1	L	No	No	5	No	No	Yes	24	Hydrochloroquine	e^b^
P2	M	50	0.5	0.5	L	Yes	No	10	No	No	Yes	24	No	f^b^
P3	M	53	5	1.5	D	Yes	Yes	14	No	No	No	58	Methotrexate	c
P4	F	41	5	4	L	No	No	5	Yes	No	Yes	31	Methotrexate	b
P5	F	52	33	0.5	L	No	No	0	No	No	Yes	24	No	d
P6^a^	F	59	22	1	L	Yes	Yes	5	Yes	No	Yes	20	No	g^b^
P7^a^	F	45	6	2	L	No	No	0	No	No	Yes	22	No	a

Disease duration is given in years. D, diffuse cutaneous skin involvement; F, female; ILD, interstitial lung disease; L, limited cutaneous skin involvement; M, male; mRSS, modified Rodnan Skin Score; PAH, pulmonary arterial hypertension; SSc, systemic sclerosis.

a Did not participate in the focus group due to sickness, but did fill in the questionnaires and made a drawing.

b Figure S1 (see Supporting Information).

### 3.2 Brief Illness Perception Questionnaire

**Figure 1:**
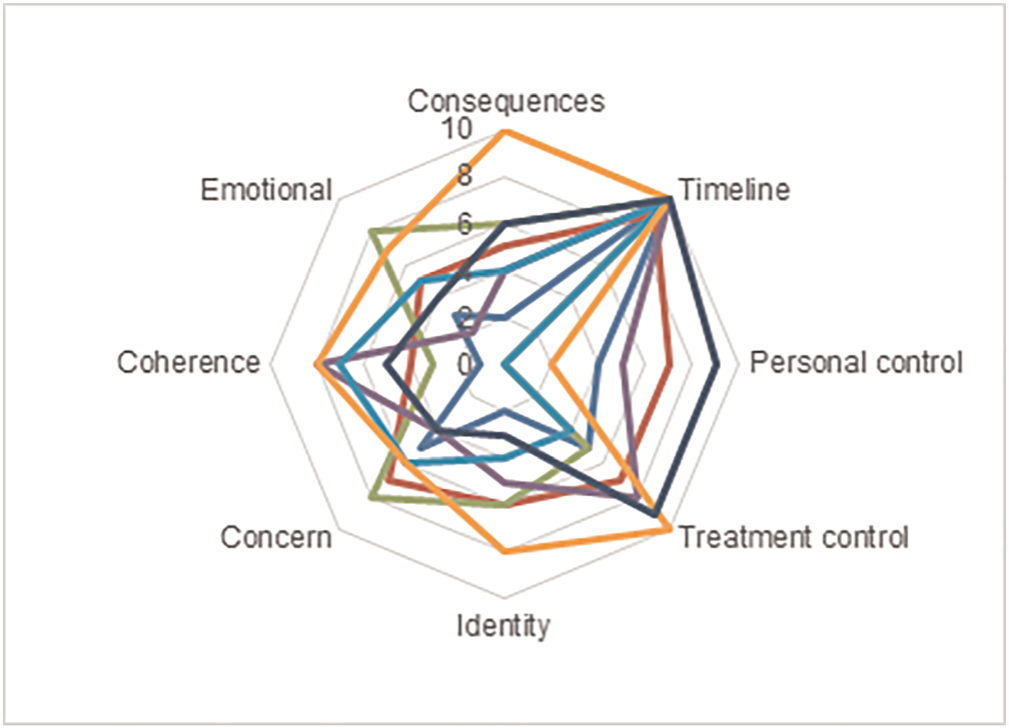
Brief Illness Perception Questionnaire dimensions. Every colour represents one patient, range 0–10 [Colour figure can be viewed at wileyonlinelibrary.com]

**Figure 2:**
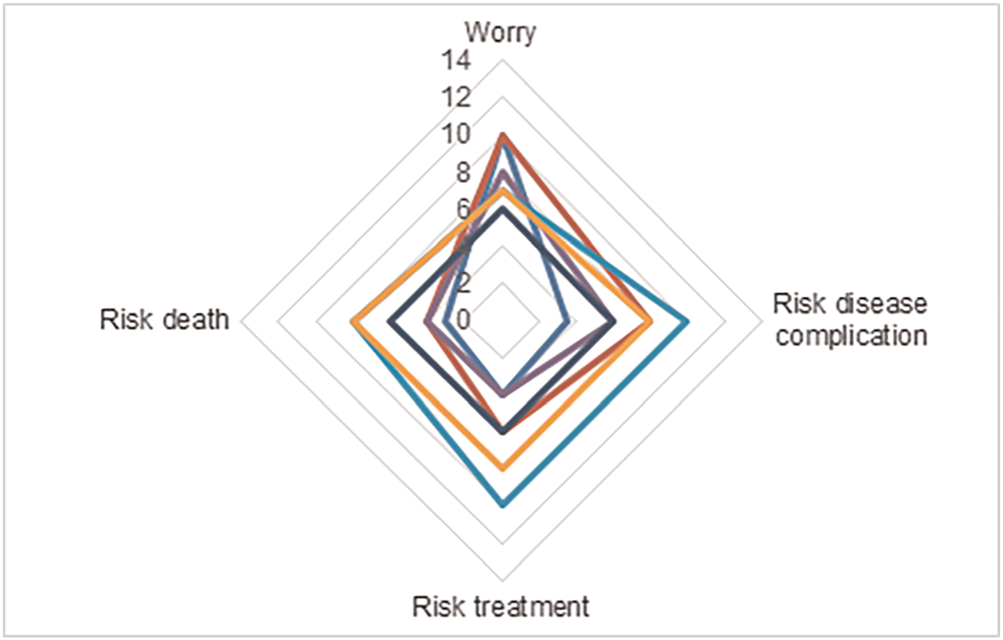
Score on worry and risk questionnaire, range 2–14. Every colour represents one patient [Colour figure can be viewed at wileyonlinelibrary.com]

Figure 1 illustrates the diversity in BIPQ scores for each illness perception per patient. The mean patients' BIPQ scores for each illness perception is shown in Table 2 (and Figure 2). The mean BIPQ score was high for timeline (mean ± SD, 9.6 ± 0.4), which indicates that the participants perceived SSc as a condition that would last forever. The participants perceived SSc as reasonably controllable with treatment (6.9 ± 2.3). As shown in Figure 1, the level of personal control varied considerably between patients (3.9 ± 3.4, range 0–10). The majority of patients felt little personal control over SSc and experienced many concerns (5.7 ± 1.5). The patient with the highest score on perceived consequences (“SSc affects my life severely”) scored highest on identity (“many severe symptoms”) and on treatment control (“treatment is extremely helpful”). Two patients with the lowest score for personal control (“absolutely no control over the disease”) both scored high on the dimension concern (“extremely concerned”) and low on the dimension treatment control (“treatment is not helpful”).

**Table 2:** Scores of BIPQ dimensions

Dimensions	Mean ± SD all 7 patients	SD	No. participants above midpoint >5 (total *n* = 7)
Consequences	5.3	2.5	3
Timeline	9.6	0.4	7
Personal control	3.9	3.4	3
Treatment control	6.9	2.3	5
Identity	4.9	2.0	3
Concern	5.7	1.5	4
Coherence	5.1	2.7	4
Emotional	4.9	2.1	3

BIPQ, Brief Illness Perception Questionnaire. Means ± SDs of BIPQ dimensions, and number of participants scoring above midpoint, range 0–10.

#### 3.2.1 Self‐reported causal perception

The most frequently mentioned causal factors for SSc were stress (*n* = 4) and genes (*n* = 3). Bad luck, menopause and heavy physical work were also causal factors mentioned by the patients.

#### 3.2.2 Worry and risk perceptions

**Figure 4:**
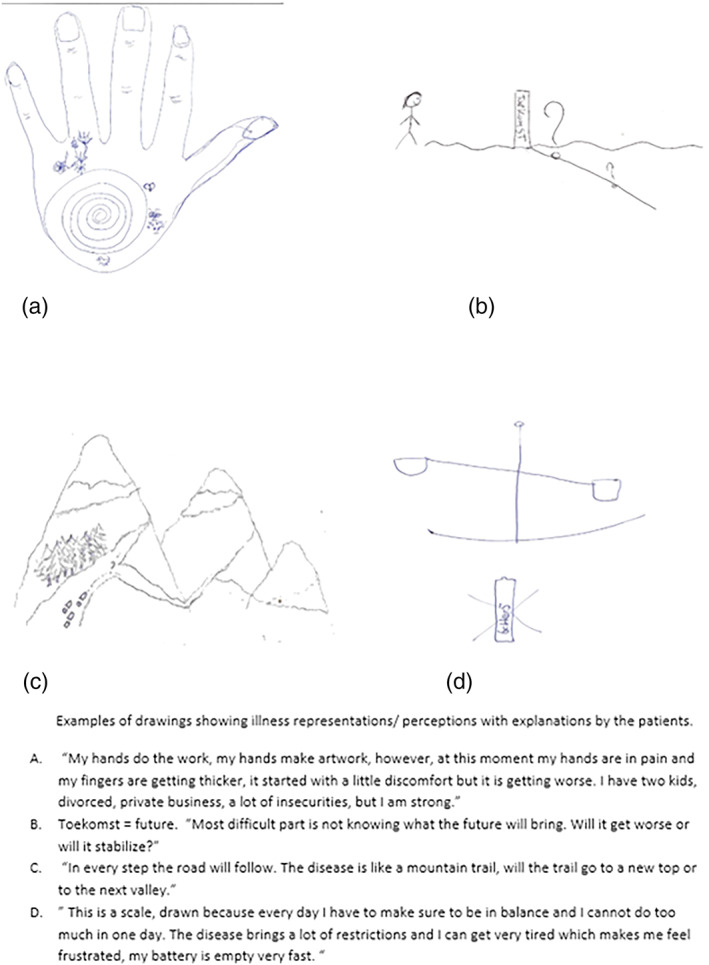
Examples of drawings showing illness representations/perceptions with explanations by the patients. (a) “My hands do the work, my hands make artwork, however, at this moment my hands are in pain and my fingers are getting thicker, it started with a little discomfort but it is getting worse. I have two kids, divorced, private business, a lot of insecurities, nut I am strong”. (b) Toekomst = future. “Most difficult part is not knowing what the future will bring. Will it get worse or will it stabilize?”. (c) “In every step the road will follow. The disease is like a mountain trail, will the trail to a new top or to the next valley”. (d) “This is a scale, drawn because every day I have to make sure to be balance and I cannot do too much in one day. The disease brings a lot of restrictions and I can get very tired which makes me feel frustrated, my battery is empty very fast” [Colour figure can be viewed at wileyonlinelibrary.com]

Worries on symptom deterioration were present in all patients, with a mean ± SD score of 7.5 ± 2.7 on a scale of 2–14 (Figure 4). The majority of patients (*n* = 6) felt they were at risk for disease complications (mean ± SD, 7.1 ± 2.7 on a scale of 2–14), which is also shown on the BIPQ dimension timeline and concern. The score for perceived risk of patients on receiving intensive treatment somewhere in the future was 6.1 ± 2.7 (*n* = 5 scored above the midpoint) and the score for perceived risk of dying due to SSc‐related complications was 4.9 ± 2.2 (*n* = 3 scored above the midpoint).

### 3.3 Focus group

Identity, consequences, personal control and concern were the illness perception dimensions mentioned most frequently. Living with SSc appears to be a dynamic process where symptoms, physical health and mental health can change daily. This process includes regaining control over personal life. Patients mentioned the following regarding personal control:

*A certain mindset is what you need, making specific things less important. [P3]*

*I changed my lifestyle to minimize the symptoms I experienced. [P4]*

*I thought I would be the one whose disease would disappear. Admitting to having a chronic disease did take a long time. [P3]*

*All my thoughts and concerns, I keep them behind closed doors and act like they do not exist. [P5]*

Some patients experienced a mismatch between their mental capacity and physical health. The majority of patients changed their lifestyle to benefit their health. In particular, patients had to change from a full‐time to a part‐time job, change to a less physically demanding job, give up or change their sport routine, sleep more hours, or make decisions about participating in activities that they took part in without issues before getting ill. The consequences of the disease were expressed in different ways:

*My husband and children live in high gear around me, and I am already glad if I can make it to first gear. [P5]*

*Every time I wear the gloves for the Raynaud Phenomenon, I feel obliged to explain this to everyone. [P1]*

*I would love to have an extra battery, or a docking station which loads my energy levels during the night. [P5]*

At start of the symptoms, the majority of patients had their symptoms dismissed or these were misdiagnosed. When a diagnosis was finally made, this brought great relief. The dimension identity came forward during the focus group in the following quotes:

*Finally hearing the diagnosis fit like a puzzle piece. [P4]*

*It [the disease] does not show on the outside. People often tell me that I look good without knowing what happens on the inside. [P2]*

*I am more tired than before, which is hard to accept. [P3]*

Many concerns were raised about the future and the disease progression, which also caused mood swings and concerns.

*I fear how the disease will evolve. [P4]*

*The disease brings a lot of insecurities, and you do not know what tomorrow will bring. [P2]*

*Which level of the disease course do you step in? The disease course can vary from mild to severe, where on this scale am I? [P4]*

Some patients described that after diagnosis, they searched for information about SSc, but that the pictures of patients and/or statistics on reduced life expectancy upset them. Most patients were displeased by reactions from their social environment. In particular, patients without visible features of SSc were frustrated by family members who told them “they were looking good” or “were doing fine”. The lack of knowledge about SSc from family members and physicians makes patients feel as though an explanation of the disease and symptoms is continuously necessary. Despite feeling unsupported by their personal environment, none of the participating patients were interested in meeting other patients in support groups.

### 3.4 Drawings

In Figure 4, four drawings are depicted (Figure S1 for additional drawings; see Supporting Information). Descriptions by participants of their drawings provide insight into how they are affected by SSc. All drawings were made in black and white. No one drew about treatments or hospital visits. The drawings include symptoms, restrictions and how these aspects affect patients emotionally. In the drawings, several dimensions of illness perception can be recognized. The most recognized dimensions are personal control (three times) and identity (three times). In most drawings more than one illness perception can be found. The portrayed hands and shoulders (Figure 4a, and Figure S1e and S1g; see Supporting Information) demonstrate which symptoms individuals associate with SSc, i.e., the illness *identity.* Three drawings also included portrayal of the participants' *concerns* regarding possible complications of SSc (e.g., Figure 4b), particularly not knowing what to expect. Interestingly, no aspect of the drawings was coded to the *timeline* item, which explores patients' perceptions of the expected duration of SSc. Some aspects went beyond existing illness perceptions. For example, some drawings showed aspects of an individual's *social environment* such as family (Figure S1f; see Supporting Information). Other aspects include activities that were restricted due to the disease (Figure 4a and 4d). Finally, drawings in Figure 4(c) and 4(d) use metaphors. The scale in Figure 4(d) stands for finding balance in life and the life metaphor in Figure 4(c) (“with each step the road becomes clear”) illustrates how this patient deals with the disease.

### 3.5 Disease severity and its association with illness perceptions, risk perceptions and worry.

The mean ± SD score for disease severity was 29.0 ± 13.2 (range in the study population 20–58 mm). The patient with the highest score on the VAS (58 mm, P3) had the lowest score on the BIPQ domain concern (score of 4) and drew Figure 4(c), which is mostly about personal control. The patient with the lowest score on the VAS (20 mm, P6) scored high on the BIPQ domain personal and treatment control (score of 9) and low on identity (score of 3), and drew the calcinosis in drawing G (Figure S1; see Supporting Information), which concerns the domain identity (symptoms). The patient (P5) with the highest score on the domains concern (score 7) and perceived consequences (score 10) and the lowest score on personal control (score 2) had a VAS score of 24 mm and drew the scale in Figure 4(d).

## 4 DISCUSSION

In this study, we explored illness perceptions, risk perceptions and degree of worry in a few representative patients with recently diagnosed SSc who had not yet developed severe complications and still had an uncertain prognosis. Our study shows that being diagnosed with SSc can have a major impact on daily life, even in an early, relatively mild disease phase, and that patients describe a broad range of illness perceptions.

The BIPQ showed that these patients believed SSc could be reasonably controlled with treatment, and that patients with a low score on personal control were hampered more by concern. The worry and risk questionnaire indicated that the majority of patients thought they were at risk for disease complications, even in this early stage of the disease. Although patients expressed loss of personal control in the BIPQ, they also described different ways of adjusting their lifestyle to regain personal control during the focus group discussions. In addition to the defined illness perceptions in the BIPQ, the drawings revealed relevant perceptions, including social environment and restrictions of specific activities. This demonstrates the additive value of the drawings, as previously described (Aminabadi, Ghoreishizadeh, Ghoreishizadeh, & Oskouei, 2011; Broadbent, Ellis, Gamble, & Petrie, 2006; Broadbent, Niederhoffer, Hague, Corter, & Reynolds, 2009).

Illness perceptions do not seem to reflect disease severity, as patients with the highest scores on identity and perceived consequences were not the patients with the most severe disease according to the physician global assessment. As illness perceptions influence illness behaviour (e.g., seeking medical help, medication adherence), it is important for physicians to be aware of this decoupling of patient perception of disease from objectifiable disease activity. For example, a patient who is short of breath might think this is just a sign of needing to rest because they perceive their current disease to be stable and mild. As such, they will not seek medical care, while in reality, this patient might be at risk for ILD progression.

To our knowledge, the BIPQ has not been used in SSc before, but some studies used the more traditional revised illness questionnaire (Moss‐Morris, Petrie, Horne, Cameron, & Buick, 2002) to evaluate illness perceptions in SSc and found that that illness perceptions were a significant contributor to physical and mental health in SSc (Arat et al., 2012; Richards et al., 2003). They also found that the unpredictable disease course and being at risk for developing disease complications were important areas of illness perceptions in these patients (Nakayama et al., 2016; Sumpton et al., 2017). The BIPQ has been used in patients with other rheumatic conditions including clinically suspect arthralgia (CSA), rheumatoid arthritis (RA) and psoriatic arthritis (PsA). In patients with CSA, identity, consequences, personal control and concern were identified as relevant, similar to what we found in patients with SSc. However, the patients with CSA more often drew the timeline dimension compared with the SSc group. This might reflect the fact that patients with CSA are at risk of developing a disease, while patients with SSc already realize the chronicity of their disease. In contrast to the patients with SSc in our study, none of the patients with CSA identified with being a patient (Newsum, van der Helm‐van Mil, & Kaptein, 2016). As SSc has the highest mortality rate among rheumatic diseases, one might expect patients with SSc to score more negatively on multiple dimensions. However, although patients with SSc showed more concern and lower personal control compared with patients with RA and PsA (Broadbent et al., 2015), they were comparable for the other dimensions. One explanation for this could be that we only included patients with recently diagnosed SSc without active severe complications to evaluate how patients deal with the diagnosis of a chronic disease with possible future disease complications. This might explain why patients with SSc score relatively low on identity (symptoms) and consequences. The fact that patients with SSc score higher on concern than patients with RA or PsA indicates that they are aware of the possible future complications. Questionnaires exploring worry and risk have not been performed before in SSc, precluding direct comparison with other studies in SSc.

Milette et al. (2019) performed a study in SSc regarding patients' perspectives on coping and disease management. The challenges discussed in that study referred to situations that hindered the possibility of coping well, including issues such as accessing information and dealing with negative emotions. We identify part of these issues in this study as well: after a diagnosis was made, patients had negative experiences caused by internet‐based information, but on the other hand felt little understanding in their personal environment. Khanna et al. (2019) showed that both internet‐based self‐management websites and educational patient‐focused books are improving self‐efficacy in patients with SSc.

Limitations of this study could be that the participants who were able to attend and participate in these focus groups represented a subgroup of patients with SSc who were potentially healthier than other patients with SSc. Furthermore, given that the patients included in this study were both willing and able to attend focus groups, this sample may also over‐represent individuals with SSc who are comfortable in participating in groups. We acknowledge that the sample size of this cross‐sectional study design is too small to provide evidence of causality. However, we aimed to explore illness perceptions and risk perceptions in early SSc and show subjective associations among the variables.

The strength of this study was the combined quantitative and qualitative analysis of illness perceptions, risk perceptions and worry in recently diagnosed patients with SSc, resulting in an unbiased approach, which has not been done before.

As shown in this study, a recent diagnosis of SSc can have a major impact on daily life and psychological well‐being even in patients with mild disease. The concerns expressed by the patients advocate for patient information and education on an individual level and in accordance with individual illness perceptions. Physicians should be aware that these illness perceptions can influence health outcomes and are not always in line with objectifiable disease measures. A multidisciplinary approach of patient‐centred care that encompasses strategies to promote self‐esteem, self‐efficacy and open communication might help to improve SSc‐related health and quality of life.

## CONFLICT OF INTEREST

All authors have declared they have no conflict of interest.

## FUNDING INFORMATION

No source of support in the form of grants or industrial support was applicable for this study.

## Supporting information


**Figure S1:** Examples of drawings showing illness representations/perceptions with explanations by the patients.
