# Supplementary figures and images for: Illness perceptions, risk perceptions and worries in patients with early systemic sclerosis: A focus group study
Source: Musculoskeletal Care. 2020 Jan 26;18(2):177–86. doi: 10.1002/msc.1453 (PMC7318332; doi:10.1002/msc.1453)

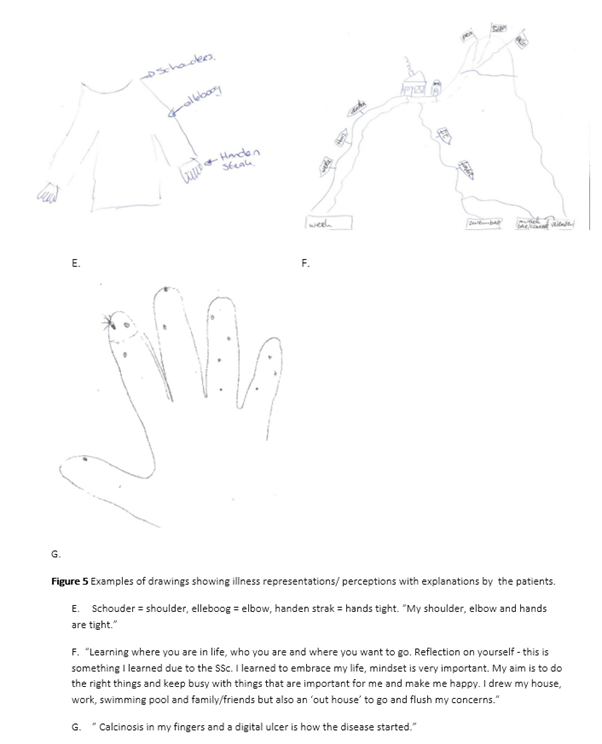

Supplement: Supplementary file 1 — Figure S1: Examples of drawings showing illness representations/perceptions with explanations by the patients. [file MSC-18-177-s001.tif]
